# Greenhouse gas emissions from global production and use of nitrogen synthetic fertilisers in agriculture

**DOI:** 10.1038/s41598-022-18773-w

**Published:** 2022-08-25

**Authors:** Stefano Menegat, Alicia Ledo, Reyes Tirado

**Affiliations:** 1 Department of Economics and Statistics, University of Turin, Turin, Italy; 2 Freelance Scientist, Huesca, Spain; 3 Greenpeace Research Laboratories, University of Exeter, Exeter, UK

**Keywords:** Environmental sciences, Planetary science

## Abstract

The global agri-food system relies on synthetic nitrogen (N) fertilisation to increase crop yields, yet the use of synthetic N fertiliser is unsustainable. In this study we estimate global greenhouse (GHG) emissions due to synthetic N fertiliser manufacture, transportation, and field use in agricultural systems. By developing the largest field-level dataset available on N_2_O soil emissions we estimate national, regional and global N_2_O direct emission factors (EFs), while we retrieve from the literature the EFs for indirect N_2_O soil emissions, and for N fertiliser manufacturing and transportation. We find that the synthetic N fertiliser supply chain was responsible for estimated emissions of 1.13 GtCO_2_e in 2018, representing 10.6% of agricultural emissions and 2.1% of global GHG emissions. Synthetic N fertiliser production accounted for 38.8% of total synthetic N fertiliser-associated emissions, while field emissions accounted for 58.6% and transportation accounted for the remaining 2.6%. The top four emitters together, China, India, USA and EU28 accounted for 62% of the total. Historical trends reveal the great disparity in total and per capita N use in regional food production. Reducing overall production and use of synthetic N fertilisers offers large mitigation potential and in many cases realisable potential to reduce emissions.

## Introduction

Food systems contribute one-third of global anthropogenic greenhouse gas (GHG) emissions, roughly 16.5 GtCO_2_e year^−1^ from a total 54 GtCO_2_e year^−1^^1,2^, with both pre- and post-production phases representing a high and increasing share of total emissions^3^. The Food and Agriculture Organization of the United Nations (FAO) estimates that agricultural emissions reached 10.7 GtCO_2_e year^−1^in 2019^4^, while the Intergovernmental Panel on Climate Change (IPCC) Special Report on Climate Change and Land^5^ estimates them at 12.0 GtCO_2_e year^−1^. In both cases, the estimated value consists of emissions from agricultural activities and land-use-related emissions. When including only the emissions up to the farm gate (excluding land use change) the total estimated by FAO reached 7.2 GtCO_2_e year^−1^ in 2019, with the principal source being livestock emissions, responsible for 51.4% of those (including enteric fermentation and manure emissions)^4^. The use of synthetic nitrogen (N) fertilisers accounted for 8.3% of farm-gate emissions in 2019^4^. When N fertiliser is applied to the soil, only a portion is uptaken by the plants. Another portion is used by soil micro-organisms, which produce N_2_O as a by-product of their metabolism while another part of the N applied may end up leaching or volatilising from the application site. Soil microbial activities release N_2_O, a GHG with 265 times more global warming potential than CO_2_over a 100 years period^5^. Reducing N_2_O emissions per unit of N compound applied is a strategy that could reduce the global impact of N fertilisers on anthropogenic GHG emissions, but the most effective strategy is reducing the N applied where over-fertilisation occurs, which is in most cases currently^6,7^. The FAO^8^ has predicted that worldwide use of synthetic N fertilisers is expected to increase 50% from the 2012 level by 2050. This will in turn lead to a large increase in N_2_O emissions from agricultural soils, potentially threatening the Paris Agreement climate target of keeping global warming within 1.5 °C or well below 2 °C above pre-industrial temperatures.

Many national GHG inventories adopt the IPCC’s Tier 1 approach (using a set of global default emission factors (EF) for different GHGs, regardless of locality). However, IPCC Tier 1 EFs have been criticised for several reasons: they assume a linear relationship between N applied and N_2_O emissions, thus ignoring non-linear dynamics and peak responses^9,10^; in the process of data collection some geographical regions are under-represented, with the EFs being mostly elaborated starting from data collected primarily in North America and Europe; and multiple crop cycles are not considered, with no account being taken of the length of data collection processes^11^. More refined EFs would ideally account for different crops, practises, soils, fertilisation regimes and regions. For these reasons, the IPCC recommends to adopt Tier 2 and 3 approaches whenever this is possible^12^. Indeed, the choice of EFs has a large impact on each country’s estimated N_2_O emissions, and thus on the estimate of the global carbon footprint of synthetic N fertilisers, which in turn may influence global GHGs accounting and finally affect efforts toward climate change mitigation.

Thomson et al.^13^ estimates that total agricultural N_2_O emissions were between 3.9 and 5.3 TgN year^−1^ in 2010, while recent data from FAO^2^ estimate them at 4.9 TgN year^−1^ in 2019. Gerber et al.^14^ estimate that in 2000 direct agricultural emissions of N_2_O from cropland were 0.66 TgN. In a more recent study, Wang et al.^15^ estimate that global N_2_O emissions from cropland reached 1.17 TgN in 2014. According to Tian et al.^16^ global anthropogenic N_2_O emissions have already exceeded the highest predictions, pointing out the importance of improving nitrogen management practises to improve farmer’s income and reduce GHG^17^.

However, these figures don't include the emissions produced by the manufacture and transportation of fertiliser products. The United Nations Framework Convention on Climate Change (UNFCCC) accounting system includes these emissions in the industrial sector rather than the agricultural sector. According to recent studies, a cross-sector approach is necessary to fully grasp the contribution of agricultural and food systems on anthropogenic GHG emissions^1,3,18^. N fertiliser production has a notable carbon footprint resulting from the fact that all synthetic N fertilisers are derived from ammonia (NH_3_) synthesised from nitrogen and hydrogen, the latter being typically obtained from hydrocarbons via the so-called steam reforming process, with associated emission of CO_2_ and methane (CH_4_). The International Fertilizer Association (IFA) reported that N fertiliser production was responsible for 0.47 GtCO_2_e and 0.93% of total anthropogenic GHG emissions in 2007^19^, a finding which drew on a previous study^20^ estimating that GHG emissions from N fertilisers production produced 0.41 GtCO_2_e in 2004. Recent estimates elaborated by the FAO indicate that in 2019 N fertiliser manufacturing accounted for about 0.41 GtCO_2_e, or 0.7% of global GHG emissions^2^.

Apart from causing the emission of GHGs in the atmosphere, the use of synthetic N fertilisers also impacts terrestrial and marine ecosystems. Mechanisms of volatilisation and redeposition of nitrogen oxides (NO_2_) may cause local off-site acidification in soils, and if treated land is adjacent to a body of water or if it is irrigated, some N may run off or leach into surface water systems. This N will ultimately end up in coastal waters, contributing to marine eutrophication^21,22^, which can have locally devastating environmental impacts.

In this study, we estimate the global climate impacts of synthetic N fertilisers, including both direct and indirect GHG emissions. Our study covers the entire product chain from manufacturing to soil application. We analyse differences between regions and countries regarding the environmental impact due to N synthetic fertiliser production and final use, in both absolute and per capita terms for the year 2018. We also underline the implications of using different emission factors (EFs) for the quantification of soil N_2_O emissions due to the application of synthetic N fertilisers, and we assess the uncertainties characterizing such emissions estimates. We complete the study with an evaluation of trends in the use of synthetic N fertilisers. This analysis helps to deepen understanding of GHG emissions resulting from synthetic N fertiliser product chains, while shedding light on the degree of uncertainty that such estimates are subject to. The study is therefore expected to advance knowledge of the climate impacts of the global food system and to help the search for effective mitigation strategies.

## N use in synthetic fertilisers

**Figure 1:**
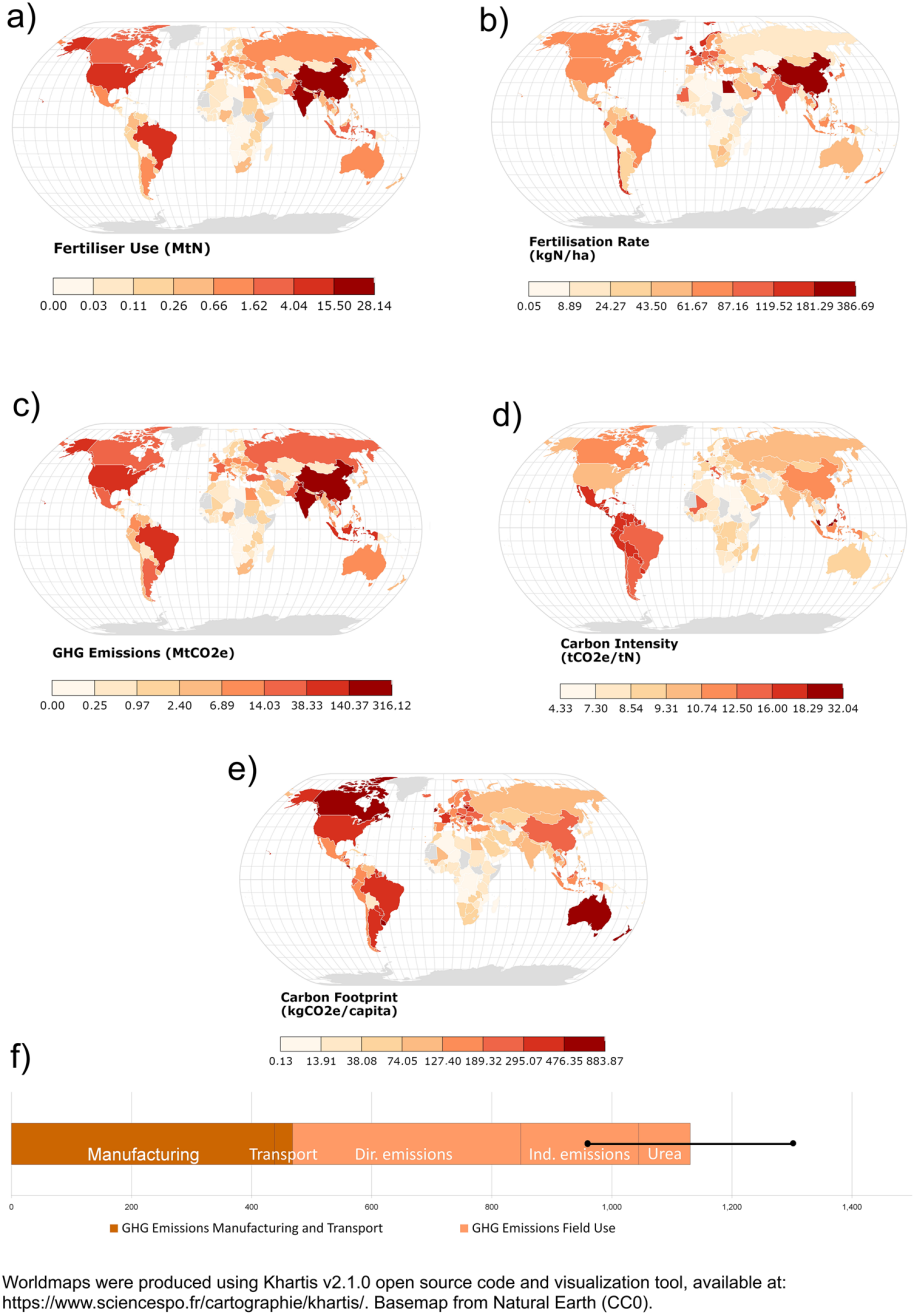
(**a**) Total amount of N fertiliser used in agriculture per country (MtN); (**b**) fertilisation rate, N fertiliser applied per hectare of cropland (kgN/ha); (**c**) Estimated GHG emissions from N fertiliser manufacturing, transportation, and field use (MtCO2e); (**d**) N fertiliser carbon intensity, GHG emissions per unit of N used (tCO2e/tN); (**e**) N fertiliser carbon footprint per capita (tCO2e/capita); (**f**) global N fertiliser GHG emissions from manufacturing, transportation, and field use (tCO2e), black bars indicate the standard deviation. Reference year for all figures is 2018.

According to FAO estimates^23^, total global agricultural consumption of elemental N from synthetic fertilisers reached 107.7 Mt in 2018, with China, India, the United States, the EU28 and Brazil accounting for 68% of the total N use (Table 1, Fig. 1a). Data from FAO also show^24^ how the countries with the highest rates of N application per hectare of cropland were located in Asia, Europe and the Middle East. On the other hand, countries in Africa tended to have among the lowest rates except in Egypt and Mauritius (Fig. 1b).According to the International Fertilizer Association^25^, 58% of global synthetic N fertiliser production in 2018 was concentrated in five countries: China, the USA, India, Russia and Indonesia, in that order. This percentage rises to 65% if the EU28 countries are included (see Supplementary Table S5). 37% of global N fertiliser production in 2018 was exported^25^, with the EU28, Russia, China, Saudi Arabia, and Qatar being leading exporters (see Supplementary Table S5). 79% of N fertiliser products were consumed within the region where they were produced, while the remaining 21% were traded across different regions^26^. All our analyses refer to N in synthetic fertilisers if not indicated otherwise.

**Table 1:** Consumption of nitrogen in synthetic fertilisers and GHG emissions from synthetic fertilisers in the world and in the top-ten emitting countries (ordered from highest total CO2e year^−1^ from N fertilisers) in 2018 based on FAO^23^ Nitrogen consumption data. Values are average ± standard deviation (SD calculated as described in methodological section).

Region	Nitrogen consumption (Mt N)	Industry emissions from synthetic nitrogen fertiliser production		Soil emissions from application of synthetic nitrogen in agriculture				Total emissions from synthetic nitrogen fertilisers (industry + agriculture)	
		Manufacturing (Mt CO_2_)	Transport (Mt CO_2_)	Urea application to soils (Mt CO_2_)	Direct N_2_O soil emissions (in Mt CO_2_e)	Indirect N_2_O soil emissions from volatilization and redeposition (in Mt CO_2_e)	Indirect N_2_O emissions from leaching (in Mt CO_2_e)	Total emissions (Mt CO_2_e)	Share of global emissions
World	107.7	438.5 ± 37.1	29.8 ± 4.0	86.0 ± 39.1	379.9 ± 160.5	66.3 ± 11.3	130.1 ± 31.4	1129.1 ± 171.1	100%
China	28.1	161.3 ± 30.1	11.1 ± 3.8	14.1 ± 6.4	73.3 ± 106.8	18.2 ± 8.8	38.1 ± 24.5	316.1 ± 113.3	28.0%
India	17.6	52.8 ± 7.9	2.4 ± 0.7	23.5 ± 10.7	51.7 ± 53.3	11.5 ± 5.6	23.7 ± 15.6	165.5 ± 57.4	14.7%
USA	11.6	40.2 ± 3.9	2.9 ± 0.7	7.5 ± 3.4	42.0 ± 51.6	7.5 ± 3.4	15.4 ± 10.0	115.5 ± 52.9	10.2%
EU28	11.1	37.5 ± 3.4	1.6 ± 0.1	5.1 ± 2.3	35.9 ± 17.5	7.2 ± 1.1	14.9 ± 3.0	102.4 ± 17.6	9.1%
Brazil	4.6	17.4 ± 1.2	2.2 ± 0.6	4.4 ± 2.0	33.2 ± 50.9	3.0 ± 1.5	6.1 ± 3.9	66.3 ± 51.2	5.9%
Canada	2.8	8.5 ± 0.8	0.7 ± 0.2	2.7 ± 1.2	15.3 ± 54.5	1.8 ± 0.9	3.7 ± 2.4	32.8 ± 54.5	2.9%
Pakistan	3.4	10.6 ± 1.6	0.5 ± 0.2	4.7 ± 2.1	10.4 ± 11.8	1.1 ± 0.9	0	27.0 ± 11.1	2.4%
Mexico	1.3	4.3 ± 0.3	0.6 ± 0.1	1.1 ± 0.5	13.5 ± 20.9	0.9 ± 0.4	1.8 ± 1.2	21.8 ± 17.5	1.9%
Indonesia	3.2	11.5 ± 1.7	0.8 ± 0.2	3.8 ± 1.7	15.8 ± 39.4	2.1 ± 1.0	4.4 ± 2.8	21.8 ± 17.5	1.9%
France	2.2	7.2 ± 0.8	0.3 ± 0.1	1.4 ± 0.6	7.2 ± 7.2	1.5 ± 0.7	3.0 ± 1.9	20.5 ± 7.6	1.8%

## Results

### Global GHG emissions associated with synthetic N fertilisers

**Figure 2:**
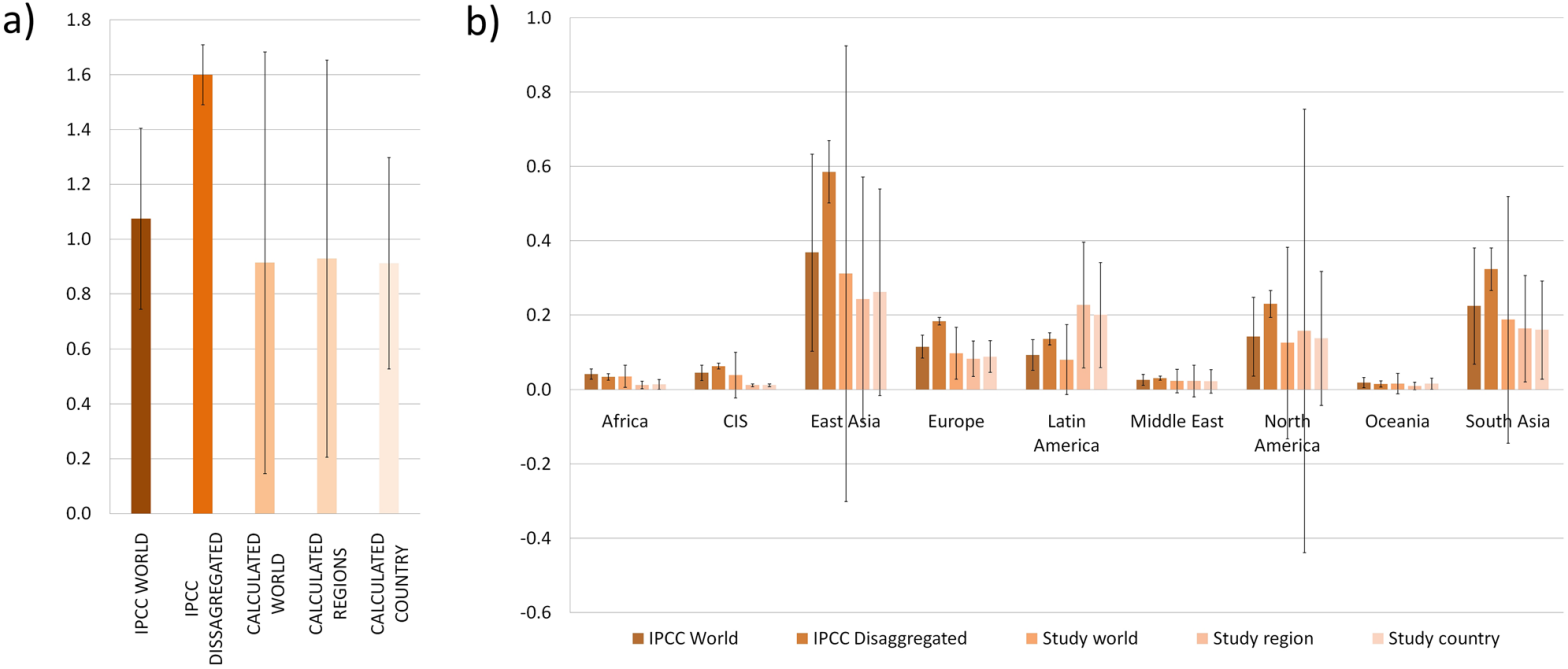
(**a**) global direct soil emissions (TgN2O) calculated using different emission factors; (**b**) regional direct soil emissions (TgN2O) calculated using different emission factors. Grey bars indicate standard deviation.

The synthetic N fertiliser supply chain was responsible for estimated emissions of 1129.1 ± 171.1 Mt CO_2_e in 2018 (mean ± sd as in Table 1 and Fig. 1f). This total value includes the GHGs emitted by fertiliser manufacturing and transportation, and the subsequent direct and indirect soil emissions resulting from fertiliser application on agricultural lands. Production of synthetic N fertilisers generated 438.5 ± 37.1 Mt CO_2_e and transportation 29.8 ± 4.0 Mt CO_2_e, while direct soil emissions added 379.9 ± 160.5 Mt CO_2_e from N_2_O emissions plus 86.0 ± 39.1 Mt CO_2_e from the CO_2_ liberated from urea. Indirect emissions resulting from fertiliser application on agricultural lands added 66.3 ± 11.3 Mt CO_2_e from N_2_O in volatilization and redeposition and 130.1 ± 31.4 Mt CO_2_e from N_2_O in leaching. The estimates of direct global soil emissions are calculated with the set of EFs that we derived from country-specific data, as we considered these to be the most accurate from the available estimations (see below and Fig. 2 for more on EFs).

In relative terms, production accounted for 38.8% of total synthetic N fertiliser-associated emissions, while emissions from agriculture fertiliser use accounted for 58.6% and transportation accounted for the remaining 2.6% (Table 1).

China was the largest emitter with 316.1 ± 113.3 Mt CO_2_e, followed by India (165.5 ± 57.4 Mt CO_2_e), and the United States (115.5 ± 52.9 Mt CO_2_e) (Table 1, Fig. 1c). The EU28 was responsible for 102.4 ± 17.6 Mt CO_2_e, thus constituting the fourth-largest emitter globally if considered as a whole. The carbon intensity of global N fertiliser supply chains, calculated by dividing the quantity of GHG generated by the quantity of N fertiliser consumed, averaged 10.48 tCO_2_e/tN, with significant differences between world regions (Fig. 1d). Thanks to their intensive use of synthetic N fertilisers and their climatic conditions, countries in Latin America tended to show a carbon intensity almost double the global mean. High carbon intensities for some European countries were the result of high soil N_2_O–N EFs, whereas in other regions such as the Middle East and East Asia the carbon intensity of synthetic N fertiliser was above the global mean because of the high carbon intensity of regional manufacturing processes^26,27^ (see Supplementary Fig. S1).

The global mean per capita carbon footprint of N fertiliser supply chains in 2018 was 148.1 kg CO_2_e cap^−1^, with significant disparities between countries (Fig. 1e). Above-average per capita footprints resulted from high fertiliser use and low population densities (North America, Oceania) and wet climatic conditions increasing the amount of N_2_O direct soil emissions (Central and South America, Europe).

### Impact of different soil EFs on global direct N_2_O emission estimates

The value of the estimates for global direct soil N_2_O emissions from synthetic N fertiliser use varied by almost a factor of 2 depending on the EF used, ranging from 379.9 ± 160.5 to 666.2 ± 45.7 Mt CO_2_e year^−1^ (from 0.91 ± 0.39 Mt N_2_O–N year^−1^ estimated with our empirical country-based EFs to 1.60 ± 0.11 Mt N_2_O–N year^−1^ estimated through the global wet-dry disaggregated IPCC EFs, Fig. 2a).Using the IPCC global EF (tier 1) resulted in an estimate of 1.07 ± 0.33 Mt N_2_O–N year^−1^, a value in line with FAO estimates adopting the same EF^28^ (Fig. 2a, see also Supplementary Table S6).

By breaking down the global direct soil emissions by region we found that East Asia was the largest contributor, with 29% of the total, followed by Latin America with 22% (Fig. 2b). Africa and the Russian Commonwealth were responsible for only around 1% of the global direct soil emissions each. In terms of individual countries, China led with 19.3% of the global emissions, followed by India (13.6%), the US (11.6%), the EU28 (9.4%) and Brazil (8.7%) (Table 1). Regardless of which set of global, regional or country level EFs was used, the regional variation pattern of estimated N_2_O emissions was similar (Fig. 2b): East Asia was the largest contributor and Africa, the Middle East and Russia the smallest.

By estimating regional EFs we found notable variation from region to region based on empirical data (Supplementary Table S7). Russian Commonwealth and Africa had the lowest EF values, with 0.3% N_2_O–N, followed by Oceania and East Asia with 0.5%; Europe and East Asia had values of 0.7%. The Americas had notably higher EFs: 1.1% and 2.4% N_2_O–N for North and South America respectively. The EFs we determined in this study for East Asia and Africa were lower than those provided by the IPCC, while the opposite was the case for Latin America. This finding suggests that earlier studies using the IPCC values may have overestimated emissions for Africa and underestimated them for Latin America. Emissions estimates for North America and Europe were more consistent among studies using different EFs, confirming the reported bias in data abundance towards those regions.

### Uncertainty around the GHG emissions estimated and variations around the EF estimated

Using the country-level EFs we estimated for direct N_2_O soil emissions and following the Monte Carlo method, we found that total GHG emissions attributable to synthetic N fertilisers had a 15.1% uncertainty (coefficient of variation). The Monte Carlo simulations showed that global GHG emissions due to the production of N fertilisers had a coefficient of variation of 8.5% while the uncertainty for the GHG emitted during transportation was 13.5%. The estimate of global soil emissions from synthetic N fertiliser showed a coefficient of variation of respectively 42.2% for direct emissions, 45.5% for urea volatilization, 17.0% for indirect emissions caused by volatilization and redeposition, and 24.1% for indirect emissions caused by leaching (Table 1).

The aggregate uncertainty was therefore heavily influenced by the uncertainty in the quantification of N_2_O soil emissions. Changing the set of EFs used to quantify N_2_O soil emissions produced estimates with significantly different uncertainties. By using the IPCC global and “wet-dry” emission factors, we found aggregate uncertainties between 12.6% and 5.7%. By using our empirical dataset to determine global and regional emission factors, we found that aggregate uncertainty was respectively 28.9% and 27.1%.

Based on our country-level EFs, we estimated that N_2_O–N direct field emissions per unit of synthetic N-fertiliser used averaged 0.0085 ± 0.0036 globally. Estimates produced with our regional and global EFs gave similar results (see Supplementary Table S7). Such global EFs for soil emissions calculated through our empirical dataset differ sensibly from the figures obtained by using the IPCC default factor (0.0100 ± 0.0031 for the aggregated method). IPCC’s disaggregated EFs might provide a more robust estimate of a global EF than the one obtained through the aggregated method. However, a rigorous estimation based on the disaggregated method would require spatially-explicit data on N fertilizer consumption, a type of information that is not currently available. The estimate we obtained through the disaggregated method, based on our assumption to assign wet and dry EFs to each country depending on the climatic condition predominant in croplands, produced a global EF 50% higher than the aggregate IPCC’s one, while the resulting uncertainty was sensibly lower (0.0148 ± 0.0010). The discrepancy between the EFs we estimated and the ones calculated following the IPCC’s method is mostly due to the fact that we collected empirical data following a bottom-up approach, which allowed us to assemble heterogeneous data with more data points (i.e. having different crops, climates and fertiliser regimens) with high spatial resolution, and therefore higher accuracy.

### Historical trends in synthetic N fertiliser use

**Figure 3:**
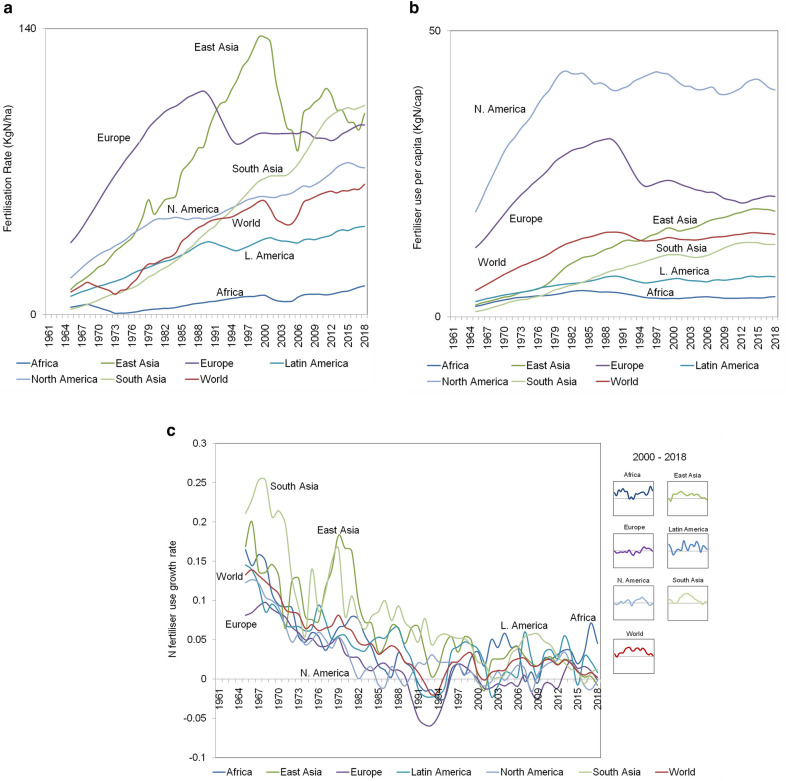
(**a**) fertilisation rate, N fertiliser applied per unit of cropland (kgN/ha); (**b**) amount of synthetic N fertiliser used (kg) per capita; (**c**) synthetic N fertiliser use: yearly growth rate for different world regions. Data source: FAOSTAT (see also Supplementary Table S4). Reference period: 1961–2018, 5-year moving averages.

Global crop production has been increasing over the past sixty years, with an annual growth rate steady at around 2.5–3% (Supplementary Table S4), but the pattern that synthetic N fertiliser use has followed over the same period is not as clear. While its use has continued to increase, the annual growth rate has fallen from 15% in the 1960s, stabilising in the last few years around 2.5%. The break point was observed between the 70 s and 1980s, when annual growth rates in Africa, Latin America, and Russian Commonwealth decreased to 5%, while they decreased to 2% in Oceania, North America and the Middle East, and around 1% in Europe, East Asia and South Asia (Fig. 3c). Interestingly, in the most recent years, the annual growth rate in synthetic fertiliser use shows a strong increasing trend in Africa (Fig. 3c).

The global mean annual per capita use of N fertilisers has increased since the Green Revolution. In 1960, mean annual use was around 3 kg N per person, but by 2018 this had risen to 15 kg N per person. Both the level of use and the rate of increase varied significantly between different regions (Fig. 3b). North America had the highest annual per capita N use value, at 40 kg, followed by Europe with 25–30 kg. In recent years, a general trend of increasing N use per capita is shown in Europe and Latin America. Africa is notably at the bottom, with annual per capita N use of around 2–3 kg, a value that has not changed over the last 60 years.

Besides being among the major factors influencing the global N cycle, fertilization rates are also determinants of N fertiliser use efficiency (NUE)^29^. Total N fertiliser applied per unit of cropland has been increasing exponentially between 1961 and 2000, with Europe peaking at about 110 kgN/ha in the 1980s, and East Asia reaching a peak at around 130 kgN/ha in the end of the 1990s. During the past two decades, the global average fertilization rate has been increasing because of growing fertilisation rates in South Asia, North America, and, to a less extent, Africa and Latin America (Fig. 3a).

## Discussion

Current projections under a baseline trajectory show that global food systems climate emissions alone could preclude achieving the Paris Agreement’s 1.5° and 2 °C targets^30^. Our analysis shows the role that the production and use of synthetic N fertilisers play in this scenario of increasing global emissions, contributing 6.8% of agri-food systems emissions annually (1.13 GtCO_2_e year^−1^ in 16.5 GtCO_2_e year^−1^).

Synthetic N fertilisers are a fundamental input in crop production^31^ and their use could increase more than predicted by FAO (about 50% between 2012 and 2050)^4^. According to estimates by Mogollón et al.^32^, N consumption in synthetic fertilisers could reach 260 Mt N year^−1^ (from 107 Mt in 2018, a 138% increase) under the most intensive scenario. On the other hand, under a sustainability scenario with NUE increase, global N use in synthetic fertilisers falls and stabilizes at 85 Mt N year^−1^ in 2050 (a 22% decrease). Only this most optimistic sustainability scenario would be within the planetary boundary established for N, which are defined as a safe operating space for ecosystems and human societies^32,33^. However, FAO has reported that between 1990 and 2019 global annual N_2_O emissions from synthetic fertilisers increased by 44%^4^.

Synthetic N fertiliser supply chains entail significant GHG emissions both from their production and from their actual use. Our estimate is based on what is a full cycle analysis of synthetic N fertilisers done with a comprehensive global methodology, an approach recently applied by both the Joint Research Centre of the European Commission^1^, and the FAO^3^. In previous studies, much more attention was given to the amount of GHG emissions arising from N fertiliser field use, while the contribution from production has received much less attention in terms of accounting, mitigation and potential efficiency gains, at least until recently. Emissions from production and transportation (0.48 GtCO_2_e) accounted for 41.4% of global GHG emissions from synthetic N fertilisers in 2018, with use-related emissions (0.66 GtCO_2_e) accounting for the remaining 58.6%. These figures represent respectively 0.9% and 1.2% (totalling 2.1%) of global anthropogenic GHG emissions, Such values are close to recent estimates made available by the FAO for 2019 (1.2 GtCO_2_e and 2.1% of global GHG emissions), although the latter do not include GHG emissions from transportation. Our results are also in line with previous estimates from the FAO^34^ (1.09 GtCO_2_e and 2.1% of global GHG emissions) and the IFA^19^ (1.11 GtCO_2_e and 2.5% of global GHG emissions). We also estimated the manufacturing climate impact of synthetic N fertiliser production by using Brentrup et al.^27^ regionalized EFs, the most recent alternative to Agri-footprint 6.0^26^ available, and our 2018 activity data. We found that the results (492 MtCO_2_e) are close to our estimate based on Agri-footprint 6.0 (438.5 ± 37.1 MtCO_2_e).

The lack of recent peer-reviewed research articles on regional emission factors for N fertiliser manufacturing is, however, an issue that merits further attention. Besides this limitation, our study relied on several methodological assumptions that were introduced to overcome the lack of spatially-explicit data on synthetic N fertiliser consumption. High resolution data on N fertiliser field-use is needed to account for both edapic and climatic conditions that would allow for a more precise estimation of global direct and indirect N_2_O emissions. Furthermore, the results of our study reflect two additional limitations that should be addressed by future research: first, the relative scarcity of empirical EFs for both direct and indirect N_2_O emissions for most countries, and second, the lack of estimates of the uncertainty characterizing activity data. While the second issue, the lack of uncertainty estimates for activity data affects field, manufacturing, and transport emission estimates equally, the lack of empirical EFs for both direct and indirect N_2_O emissions for most countries is a major source of uncertainty for field emissions.

Indeed, estimates of GHG emissions from N fertiliser use exhibit larger uncertainty than from production and transport (Table 1, Fig. 2). Our estimates of N_2_O direct soil emissions from applied fertiliser had uncertainties of 84.1% for the global value and 77.9% for the regional averages (Fig. 2). The processes and patterns governing N_2_O emission after N fertiliser application are complex and not fully understood. Besides, the lack of empirical studies recording long-term N_2_O emissions from different agricultural practices and crops in different world regions precludes the availability of accurate EFs. Moreover, such EF data are over-representative of field studies in North America and Europe^11^. The selection of an appropriate EF is key: as an illustration, we obtained a global value of annual N_2_O soil emissions following synthetic N fertiliser application of 0.91 ± 0.38 GtN_2_O–N using an EF which we calculated from country-specific data. However, we obtained higher estimates of 1.1 ± 0.33 GtN_2_O–N using the IPCC Tier 1 global EF, an EF that recent Tier 3 estimates^35^ have found to be representative of the global average. Clearly the difference between the highest and lowest of these estimates (Fig. 2), represents a very significant quantity of GHGs which we may be either ignoring or over-counting. Our estimate based on our own EF, though, agrees with the results of other studies^14,15^. A second source of divergence when estimating the carbon footprint from N_2_O is the global warming potential (GWP) factor used. In 2019 the IPCC revised its value to 268, down from its original (2006) estimate of 298. While we have used the updated value, studies published before 2019 used the original one.

In spite of the uncertainty in quantifying agricultural emissions of N_2_O, its share of total global GHG emissions is expected to grow in the forthcoming decades^30^. Although emissions from industry and transport could decrease in response to climate-oriented policies, GHG emissions are expected to increase if N fertiliser consumption will keep increasing. Three factors stand out as key contributors to the scale of anthropogenic losses of reactive N to the environment: the model of agriculture based on the use of synthetic fertilisers, the decoupling of arable and animal agriculture, and the growth in industrial animal production and consumption globally^7,36^. Reverting these offers the largest potential to mitigate agricultural emissions.

Considering the full N cycle, a recent study showed that NUE of crop, fodder, and forage averages 43% globally^37^. Further, Billen et al.^36^ indicate that only 20–30% of N inputs are used to fertilise crops for human food. Excessive input of N fertiliser results in more than half of applied N fertiliser being lost into the environment^7^, with very significant economic and environmental costs. While maximising NUE does not always result in minimising GHGs from agriculture^38^, increased NUE is often a good indicator of improved practices and of associated reduced emissions.

Reducing overall production and use of synthetic N fertilisers offers large mitigation potential and in many cases highly realisable potential to reduce N_2_O emissions. Mitigation of production emissions may be especially significant given the high carbon intensity of the production processes and their large contribution to overall emissions.

The management of cropland nutrients could reduce N_2_O emissions by 0.03–0.7 GtCO_2_e year^−1^ by 2050 (as estimated by Roe et al.^39^ and reported by the IPCC^5^) representing between 0.17 and 4.1% of the total potential land-sector mitigation contribution. The upper limit of 0.7 GtCO_2_e year^−1^ is estimated under a scenario of better nutrient management and a 32% reduction in synthetic fertiliser use compared to the baseline scenario. For cereal crops, Mueller^40^ calculated a high potential efficiency gain, finding that a 48% reduction in synthetic fertiliser use could be achieved with no reduction in yields.

Over-application of fertiliser is a common practice in agriculture globally, with damaging consequences beyond the GHG emissions that we have focused on in our analysis. Improving farm practices to ensure improved N management, especially among smallholders, will not only be beneficial for their economic returns and for the climate, but will also reduce other environmental and health impacts (e.g. nitrate pollution, acidification, eutrophication). For example, Gerber et al.^14^ estimated that reducing N application rates only by 5% in China’s Shandong province, would reduce the province's share of global N_2_O emissions by 9% and global crop N_2_O emissions by 0.35%.

In addition to cutting excessive synthetic N fertiliser use, many agronomic practices work synergistically with soil microbiota to reduce the need for N application and/or reduce N losses from soils. For example, the use of green manures with N-fixing crops not only reduces the need for N application but improves soil structure and water retention capacity while reducing erosion—all helping to reduce leaching and runoff^41^.

Replacing synthetic N fertiliser with organic N fertilisers may offer significant potential for emissions mitigation in places where excess N is available to be applied to agricultural land. Enormous amounts of N are present in organic wastes (e.g. manure from animals, domestic sewage); however, the feasibility of using such sources needs further attention, as it holds many practical difficulties^42^^.^

Globally, livestock produced 125 TgN year^−1^ in manure in 2018^43^, which was roughly similar to the total amount of N in synthetic fertilisers applied to soils, 107 TgN in the same year. However, estimates indicate that less than 50% of N excreted by livestock is returned to agricultural land, and even then a large part of this N is subsequently lost to the environment^42,44^. According to recent estimates^45^, only 22 TgN of livestock manure were used to fertilise crops in 2018. Increased recycling of livestock manure nutrients to cropland could replace synthetic fertiliser nutrients, at least in part, offering a strategy to mitigate GHG emissions^42,46^. Nevertheless, there are questions over the relative efficacy of manure and synthetic fertilisers, which merits further research^47^.

The large contribution of synthetic N fertilisers to global GHG emissions needs to be seen within an overall framework of agricultural and food systems sustainability. Ending excessive N application and increasing the recycling of N within agricultural systems should be established as basic principles of such a framework, together with the reduction of industrial animal food production (as the main contributor to GHG emissions from agriculture globally).

Our study reflects three important realities. First, the estimation of GHG emissions from synthetic N fertiliser still holds large uncertainties, especially where emissions from use are concerned. This issue should be addressed as soon as possible, not least to improve estimates of emissions from different fertilisers as well as their use on different crops in different world regions (especially Asia—the world leader in synthetic N fertiliser use—and Africa, about which there is currently little data). Robust data will be key to the development and monitoring of the climate mitigation programmes^45^.

Secondly, the large impact of synthetic N fertiliser on climate emissions requires the development of a comprehensive scheme to reduce its overall use and increase efficiency of N recycling in agricultural and food systems. This is a complex challenge, since different and unrelated actors are responsible for emissions in different parts of the system (for example, emissions from synthetic fertiliser production depend on the industrial sector, N_2_O emissions from applied fertiliser depend on farming practices, while the food demand and dietary patterns that drive these emissions depend on human populations and cultures).

Thirdly, while food production is not expected to decline in a growing global population scenario, enough food to feed a growing population could be produced with a much smaller contribution to global GHG emissions, without compromising yields or food security^14,36^. Shifting dietary patterns towards less meat and dairy products could play a central role, since the increasing share of animal products in protein nutrition per capita is the key driver of the agricultural production system. Three quarters of N in crop production (expressed in terms of protein and including bioenergy by-products) is currently devoted to livestock feed production globally^7^.

To conclude, there is no doubt that emissions from synthetic N fertilisers need to be reduced (instead of increasing as predicted under current trajectories), if the goal of keeping global heating within 1.5 °C of pre-industrial levels is to be achieved.

## Methods

Collection of the data necessary to perform our analysis involved three main steps. First, we created a database of information on global agricultural use of synthetic N fertiliser (activity data), using the FAO definition of agricultural use, which refers to use for crops, livestock and forestry (see FAOSTAT for details). Second, we retrieved emission factors (EFs) for N fertiliser manufacturing, transportation, and field emissions from the literature and from field experiments. Third, we calculated the amount of CO_2_e emissions attributable to the three stages of the synthetic N fertiliser product chain: (i) the production of N fertiliser products, (ii) their transportation from the factory to the field, and (iii) their soil emissions from use in agriculture.

### Activity data

Country-level data for agricultural use of synthetic N fertilizers per country and year were retrieved from FAOSTAT (http://www.fao.org/faostat/en/#data). Total agricultural consumption of N in synthetic fertilisers was 107 Mt in 2018, the most recent year for which annual data was available at the time of this study. We used this value to estimate GHG field emissions. 96% (102 Mt) of the synthetic N consumed globally was supplied in the form of eight fertiliser product groups that we took as the cornerstone of our estimate of both manufacturing and transport emissions. The product groups considered included anhydrous ammonia, ammonium nitrate (AN), ammonium sulphate (AS), calcium ammonium nitrate (CAN), urea, nitrogen solutions (UAN), ammonium phosphates (AP), nitrogen, phosphorous, and potassium compounds (NPK) (see Supplementary Table S1). The remaining 4% for which no detailed information was available (i.e., fertiliser product type, emission factors, and trade data) has been included in the estimate of direct and indirect field emissions but it could not be counted in our estimate of the GHG emissions due to N fertiliser production and trade, and hence our results underestimated global emissions. To estimate the amount of GHG emissions due to urea hydrolysis, we collected activity data relative to national consumption of both urea and UAN (urea solutions) and converted them from N-base to product-base through the following steps: first, we converted the amount of urea consumed per country from a nutrient base (tonnes of N) to a product base (tonnes of product) by dividing the nutrient-base value by 0.466 (nitrogen content of urea). We also converted UAN from a nutrient-base (tonnes of N) to a product base (tonnes of product) by dividing the nutrient-based value by 0.30 (nitrogen content of UAN). We calculated the amount of urea contained in the amount of UAN used by each country by multiplying the amount of UAN used by 0.35 (average content of urea in one unit of UAN). Finally, we summed up urea (tonnes) and urea-equivalent UAN (tonnes) to obtain total urea consumption (tonnes).

The product breakdown and the list of countries by regional classification were retrieved from IFASTAT (https://www.ifastat.org/). Our choice to use IFASTAT instead of FAOSTAT product breakdown data in our dataset was motivated by the fact that detailed data on N fertilizer products for China “mainland” was not available in FAOSTAT^48^. Since China is the most important consumer of synthetic N fertilizer globally, we decided to adopt IFASTAT’s estimate^25^. Using IFASTAT's country-level market mixes and FAOSTAT's data on national consumption of synthetic N fertiliser, we estimated the amount of each fertiliser product (*j*) consumed in each country (*i*) in 2018. We didn't include an uncertainty assessment of the activity data because of a lack of information (IFASTAT, personal communication). For what pertains the amount of GHG emissions estimated through the IPCC disaggregated (“wet-dry”) approach, we relied on a country-base climatic classification that might lead to overestimating direct and indirect soil N_2_O emissions. Because no spatially-explicit data on synthetic N fertiliser consumption is available, we assigned to each country a predominant climatic classification (either “wet” or “dry”) based on the distribution of the cultivated land throughout different climatic zones.

The dataset assembled for this study is available in the supplementary materials (Supplementary Table S2). To calculate the amount of GHG emissions generated through the processes of synthetic N fertiliser production, transportation and agricultural use, we followed the IPCC guidelines for national GHG inventories^12^. We accordingly calculated the emissions at country level on the basis of the activity data we had collected and the appropriate EFs.

### Emission factors

#### EFs for manufacturing and transportation

We used the Agri-footprint 6.0^26^ dataset to calculate the EFs for both manufacturing and transportation of different synthetic N fertiliser products. Agri-footprint 6.0, which relies on an extended version of the European Platform on Life Cycle Assessment’s European reference Life Cycle Database (ELCD), provided us with the regionalised EFs both at the factory gate and at the final market of consumption (thus including the emissions due to the transportation from the factory to the region of consumption) of eight fertiliser products.

For what pertains to the emissions at the factory gate, Supplementary Fig. S1 provides the complete list of the products included in the Agri-footprint 6.0 dataset and the regional EFs expressed as relative to the European (RER) average. Absolute values of Agri-footprint 6.0 EFs may be made available by Blonk Consultants on request. Except for a few discrepancies, Agri-footprint 6.0 EFs align quite well with the EFs elaborated by Brentrup et al.^27^, the ones also adopted by FAOSTAT^2^. Supplementary Fig. S1 also shows the similarities and discrepancies between Agri-footprint 6.0 and Brentrup et al.^27^ datasets, focusing on how regional EFs compare to the RER average of each dataset. Since IFASTAT only provides data for Ammonium Phosphates (without specifying between Mono-ammonium Phosphate and Di-ammonium Phosphate), we applied only the Mono-ammonium Phosphate EFs for both manufacturing and transportation. EFs for urea manufacturing did not include the amount of CO_2_ incorporated in the manufacturing process and released on the field via hydrolysis. We estimated the uncertainty in the EFs for all products by assuming a standard deviation of 25%, a lognormal probability density function^49,50^, and maximum and minimum values as presented in Kool et al.^51^.

For what pertains transportation, Agri-footprint provided us with data detailing: (i) the region of manufacturing and the region of consumption of each fertiliser product, (ii) the average distance travelled by each N fertilizer product both within the region of use and between other regions and that region, and (iii) the mix of transport modes characteristic of domestic, regional and inter-regional transportation of fertilisers. Based on this, we calculated the EF for the transportation of a unit of each fertiliser product within a given region and between regions. We estimated the uncertainty of the EFs by assuming a standard deviation equal to 50%, and a normal probability density function.

#### EFs for direct field emissions

The EFs for the direct and indirect emissions taking place at the field level were retrieved or estimated through different sources in order to compare different possible scenarios and estimate national, regional, and global emissions of N_2_O–N due to synthetic N fertilisers field use.

For what pertains to direct field emissions of N_2_O–N, we retrieved three sets of EFs: the first set included the IPCC's^12^ global EF of 0.010, with an uncertainty range 0.001–0.018. We assumed that 0.001 was the minimum value, 0.018 the maximum value, and 0.009 the standard deviation. The second set included the disaggregated IPCC's^52^ EFs for dry and wet climates, where wet climates had an EF equal to 0.016 and a standard deviation of 0.003 (we assumed a minimum value of 0.013 and a maximum value of 0.019), and dry climates had an EF equal to 0.005 and a standard deviation of 0.006 (we assumed a minimum value of − 0.001 and a maximum value of 0.011).

The third set of EFs was elaborated by ourselves and it included (a) a global EF, (b) regional EFs and (c) country-level EFs. In order to calculate our EFs, we first created a dataset of empirical observations from field experiments and/or controlled environments containing information on N_2_O–N emissions directly measured on the field in paired plots, which implies, for every location N_2_O–N emissions were measured in a plot in which no fertiliser was applied and in a similar plot (same crop, location and environmental and growing conditions) in which N fertiliser was applied. This was required so that natural emission levels could be subtracted from the overall measured fluxes from the plots where fertiliser had been applied in order to give the emission level attributable to fertiliser application. The dataset was created by combining three existing datasets: the global CGIAR N emissions dataset (https://samples.ccafs.cgiar.org/n2o-dashboard/), the tropical database published by Albanito et al.^11^ and the Chinese dataset published by Yue et al.^10^. (By the time the present study is published, the CGIAR database may have been updated to include all the data from the tropical and Chinese datasets). We removed duplicated entries and harmonised the data. We kept only entries referring to application of synthetic N fertiliser, removing data for locations with, organic fertiliser, or a mix of organic and synthetic fertilisers had been applied, or when no fertiliser was applied in any of the paired plots. The final database contained a total of 1602 empirical paired-plots measurements, The dataset is reproduced in Supplementary Table S3. We calculated country-level EFs by means of the formula (1):

1 $$EF={{{N}_{2}O}_{t}}_{i}-{{{N}_{2}O}_{c}}_{i},$$

where N_2_Oti is the value of total soil N_2_O–N emissions measured from plots where synthetic N fertiliser was applied and N_2_Oci is the value of the total soil N_2_O–N emissions measured in the control paired plot. We then estimated total N_2_O–N emission levels from synthetic N fertiliser application at country level. In the absence of other information, we assumed that empirical data points are representations of the country’s common crops and agricultural practices. In addition, we also calculated regional and global EFs. The regional EFs were calculated using country level data: we calculated the regional value as the mean of the national EFs weighted according to the number of points per country. The global EF was calculated using the mean of regions weighed by the countries per region. We estimated the uncertainty in our EFs by directly calculating the mean and the standard deviation and by assuming a lognormal probability density function, consistently with Cowan et al.^53^.

#### EFs for indirect field emissions

Following the 2019 Refinement to the 2006 IPCC Guidelines for National Greenhouse Gas Inventories (chapter 11)^52^, we calculated indirect N_2_O–N emissions from both volatilisation and redeposition (N_2_O(G)) and Nitrogen Leaching and Runoff (N_2_O(L)) using the disaggregated (wet-dry) method. For Nitrogen Leaching and Runoff (N_2_O(L)) we used the IPCC "FRACLEACH" factor (assuming a s.d. equal to 0.05) to calculate the amount of N leaching and running off and the "EF5" to calculate the amount of N_2_O–N generated (to estimate the uncertainty we assumed normal probability density function). This approach differs from the one adopted by FAOSTAT^28^, which provides estimates of N_2_O indirect emissions calculated through the IPCC’s aggregated (global) EFs. For volatilisation and redeposition we used the IPCC "FRACGASF" factor (assuming a s.d. equal to 0.05) to calculate the amount of N volatized and the "EF4" to calculate the amount of N_2_O–N generated (to estimate the uncertainty we assumed a normal probability density function).

#### EFs for urea hydrolysis

Following IPCC^12^ guidelines we calculated the amount of CO_2_–C generated by urea application of fields, by multiplying the amount of urea-equivalent applied (tonnes of product) by the given emission factor (0.2 ± 0.1), and we assumed a normal probability density function to perform an uncertainty analysis.

### Estimation of GHGs from synthetic N fertiliser production, transportation and agricultural use

To estimate global GHG emissions due to N fertiliser production, transportation, and field use, we converted the emissions generated during each phase to tCO_2_e by using the appropriate conversion factor.

Activity data on N fertiliser consumption available at the country level were multiplied by Agri-footprint 6.0 regional EFs that we assumed to be equal to national EFs. GHG emissions (*GHG*_*M*_ in tCO_2_e) for each country (*i*) were accordingly estimated through a consumption-based formula (2):

2 $${GHG}_{M,i}=\sum \left({A}_{M,j,i}\times {EF}_{M,j,k}\right),$$

where *A* is the activity data (synthetic N fertiliser consumed), *EF* is the emission factor, *j* is the type of product consumed, and *k* the region of production.

Global GHG emissions due to the production of N fertilizers were then estimated by summing all the national estimates. Next, GHG emissions due to the transportation of N fertilisers from the factory to the country of consumption were estimated using the Agri-footprint 6.0 methodology. First, we retrieved regional market mixes for the eight N fertiliser product groups from Agri-footprint 6.0. Second, by assuming that regional data were representative of national market mixes, we estimated for each country the amount of N fertiliser sourced domestically or within its own region and the amount imported from other regions. Third, we multiplied these amounts by the regional EFs described in the previous section. Country level GHG emissions due to N fertiliser transportation (*GHG*_*T,i*_ in tCO_2_e) were therefore calculated through the following formula (3):

3 $${GHG}_{T,i} = \sum ({A}_{T,j,i}\times {EF}_{T,j,k}).$$

Next, we estimated emissions resulting from synthetic N fertiliser application, including both the N_2_O–N produced from the N that is not absorbed by plants and is metabolized by soil micro-organisms, and the CO_2_ emitted when urea fertilisers are used. We followed the IPCC method to estimate N_2_O–N direct emissions. This requires the use of a N_2_O–N EF to estimate soil emissions rather than directly measuring gas fluxes. We therefore calculated GHG direct emissions (*GHG*_*D,i*_ tCO_2_e) due to N fertiliser field use through the following formula (4):

4 $${GHG}_{D,i} =[({A}_{D,i}\times {EF}_{T,i,k})\times \frac{44}{28}]\times 265,$$

where 44/28 is the factor used to convert tN_2_O–N to tN_2_O, and 265 is the GWP factor (over a 100-year time horizon) used to convert tN_2_O to tCO_2_e^5^.

To estimate indirect emissions due to volatilization and redeposition (*GHG*_*V,i*_ tCO_2_e), we used the volatilization factor (*VF*) and the EF provided by the IPCC, then we converted the estimated emissions from tN_2_O–N to tN_2_O, and from tN_2_O to tCO_2_e through the following formula (5):

5 $${GHG}_{V,i} = \{[\left({A}_{D,i}\times {VF}_{V,i}\right)\times {EF}_{V,i})]\times \frac{44}{28}\}\times 265.$$

To estimate indirect emissions due to leaching (*GHG*_*L,i*_ tCO_2_e), we used the leaching factor (*VF*) and the EF provided by the IPCC, then we converted the estimated emissions from tN_2_O–N to tN_2_O, and from tN_2_O to tCO_2_e through the following formula (6):

6 $${GHG}_{L,i} = \{[\left({A}_{L,i}\times {VF}_{L,i}\right)\times {EF}_{L,i})]\times \frac{44}{28}\}\times 265.$$

Following IPCC (2006) guidelines we calculated the amount of CO_2_–C generated by urea application on fields, by multiplying the amount of urea-equivalent applied (tonnes of product) by the given emission factor. To estimate the GHG emissions due to urea and UAN field use (*GHG*_*U,i*_ tCO_2_e), we used the following formula (7):

7 $${GHG}_{U,i} = \left({A}_{U,i}\times {EF}_{U,i}\right)\times \frac{44}{12},$$

where 44/12 is the IPCC factor to convert CO_2_–C to CO_2_e. National GHG emissions due to N fertiliser manufacturing (*GHG*_*i*_ tCO_2_e), transportation, and field use were therefore estimated through the formula (8):

8 $${GHG}_{i} = {GHG}_{M,i}+{GHG}_{T,i}+{GHG}_{D,i}+{GHG}_{V,i}+{GHG}_{L,i}+{GHG}_{U,i},$$

whereas global emissions (*GHG*_*global*_ tCO_2_e) were calculated by summing up national estimates (Eq. 9):

9 $${GHG}_{global} =\sum {GHG}_{i}.$$

In aggregating national and global estimates, we computed the overall uncertainty by applying the Monte Carlo method, as suggested by the IPCC^12^. We therefore randomly generated 5000 values for each EF above described according to the uncertainty parameters discussed in the previous section, and we calculated the uncertainty for national, regional, and global estimates.

### Other indicators used in the study and historical trends of N use

By using FAOSTAT country-level 2018 data for agricultural land and population we calculated respectively the fertilisation rate (kgN/ha) and the per capita carbon footprint (kgCO_2_e/person) of each country, the latter being based on the aggregated emissions generated by the production, transportation and use of the synthetic N fertilisers consumed in the country. Similarly, we calculated the carbon intensity (tCO_2_e/tN) of N fertiliser supply chains by dividing the total GHG emissions generated (including by production, transportation and use) by the total amount of N consumed in the form of synthetic N fertiliser.

We evaluated historical trends of population (number of people), N applied in the form of synthetic N fertiliser (t), and N from synthetic fertiliser applied per unit of cropland (t/ha) covering the period 1961–2018 and using national values provided by FAOSTAT to compute aggregate values for the regional classification adopted in this study (the dataset we used is reproduced in Supplementary Table S4).

Using the above data, we calculated the annual growth rates of population, crop production, harvested area and N use in the form of synthetic fertilisers at a global scale from 1961 to 2018 according to the following formula (10):

10 $${growth \; rate}_{10years}=\left(\frac{{V}_{{t}_{1}}}{{V}_{{t}_{0}}}-1\right)\times 100,$$

where “*V*_*t*1_” is the value of the variable observed at the time 1, and “*V*_*t0*_” is the value of the same variable at the time 0. The results enabled not only the evaluation of global trends but also comparisons between world regions.

Some small countries were missing data for one or more of the variables for some years. The missing information was of minor significance, and accordingly we did not carry any gap-filling analysis.

## Supplementary Information

Supplementary Information.

## Data Availability

The datasets generated during and/or analysed during the current study are available in the figshare repositories, https://doi.org/10.6084/m9.figshare.16794115 and https://doi.org/10.6084/m9.figshare.16796569. The data relative to the emission factors for N fertiliser manufacturing and transportation are available from Blonk Consultants but restrictions apply to the availability of these data, which were used under license for the current study, and so are not publicly available. Data are however available from the authors upon reasonable request and with permission of Blonk Consultants.
