# Author Correction: Greenhouse gas emissions from global production and use of nitrogen synthetic fertilisers in agriculture

**DOI:** 10.1038/s41598-022-24242-1

**Published:** 2022-11-17

**Authors:** Stefano Menegat, Alicia Ledo, Reyes Tirado

**Affiliations:** 1 Department of Economics and Statistics, University of Turin, Turin, Italy; 2 Freelance Scientist, Huesca, Spain; 3 Greenpeace Research Laboratories, University of Exeter, Exeter, UK

Correction to: *Scientific Reports* 10.1038/s41598-022-18773-w, published online 25 August 2022

The original version of this Article contained errors. In Figure 3a and b, “KgN” in the y-axis label was incorrectly given as “tN”. The original Figure 3 and the accompanying legend appear below for the record.

**Figure 3:**
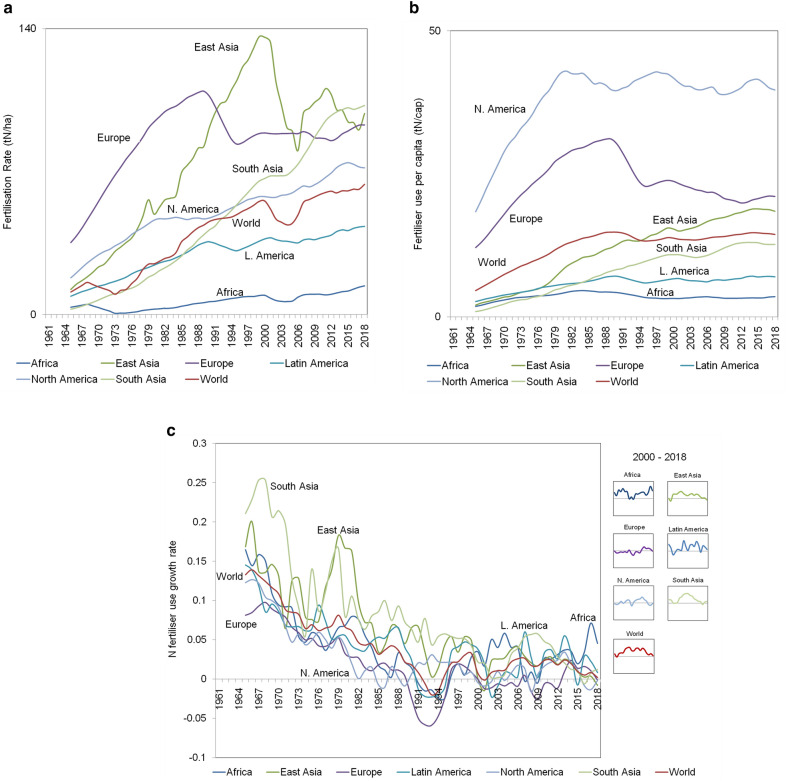
(**a**) Fertilisation rate, N fertiliser applied per unit of cropland (kgN/ha); (**b**) amount of synthetic N fertiliser used (kg) per capita; (**c**) synthetic N fertiliser use: yearly growth rate for different world regions. Data source: FAOSTAT (see also Supplementary Table S4). Reference period: 1961–2018, 5-year moving averages.

In addition, the Supplementary Information contained errors, where the values in the column "N fertiliser use" in Supplementary Table S4 were incorrect.

The original Supplementary Information file is provided below.

The original Article and its accompanying Supplementary information have been corrected.

## Supplementary Information

Supplementary Information.

